# Scrub Typhus in Mainland China, 2006–2012: The Need for Targeted Public Health Interventions

**DOI:** 10.1371/journal.pntd.0002493

**Published:** 2013-12-26

**Authors:** Wen-Yi Zhang, Li-Ya Wang, Fan Ding, Wen-Biao Hu, Ricardo J. Soares Magalhaes, Hai-Long Sun, Yun-Xi Liu, Qi-Yong Liu, Liu-Yu Huang, Archie C. A. Clements, Shen-Long Li, Cheng-Yi Li

**Affiliations:** 1 Institute of Disease Control and Prevention, Academy of Military Medical Science, Beijing, P.R. China; 2 Chinese Center for Disease Control and Prevention, Beijing, P.R. China; 3 School of Population Health, Infectious Disease Epidemiology Unit, The University of Queensland, Brisbane, Australia; 4 Chinese People's Liberation Army General Hospital, Beijing, P.R. China; University of Texas Medical Branch, United States of America

## Introduction

Scrub typhus is a vector-borne disease carried by the chigger mite. The aetiological agent is the rickettsia *Orientia tsutsugamushi*, which is endemic to several countries in the Asia-Pacific region, including China [1]. It is also a travel-associated disease [2] and of great importance among military personnel [3], [4]. During the Second World War, scrub typhus was associated with a higher case fatality ratio than any other infectious disease in the China-Burma-India theatre of operations 1,3. Clinical presentation in patients varies from asymptomatic to life-threatening disease [5], including acute hearing loss and multiple organ failure [6], [7]. To date, there is still no effective and reliable human vaccine against scrub typhus and no point-of-care diagnostics available [1].

In China, scrub typhus is primarily transmitted by the larvae of the chigger mites *Leptotrombidium deliense* and *L. scutellare*, which usually feed on rodents, including *Rattus* spp. *(R. losea*, *R. flavipectus*, *R. norvegicus*, and *R. confucianus), Apodemus agrarius*, and *Suncus murinus* [8], [9]. Humans become infected when they are exposed to areas where the chigger-infected rodent cycle occurs: for example, during agricultural and recreational activities. Excessive deforestation, changing patterns of agricultural activities, unplanned exploration of natural resources, and climate change may increase the transmission and expansion of the hosts and vectors of scrub typhus [10], [11]. For the purpose of more efficient, targeted surveillance and control for scrub typhus, it is important to investigate the spatiotemporal epidemiological pattern of outbreaks in China.

Historically, scrub typhus was first reported in China in Guangzhou (Guangdong Province) in 1948 [9]. In recent years, local outbreaks of scrub typhus have occurred in many areas of China, and recently identified endemic foci of scrub typhus are reported to be expanding [8], [9]. However, the nationwide characteristics of scrub typhus epidemics are unclear and their spatiotemporal patterns need to be investigated. Spatial epidemiological tools have been widely used to identify spatiotemporal clustering, determine important environmental drivers and predict the distribution of a range of infectious diseases [12]–[14]. A detailed understanding of China's scrub typhus epidemics is needed to develop more effective public health responses to scrub typhus outbreaks.

## Epidemic Characteristics and Spatiotemporal Pattern of Scrub Typhus in China

To describe epidemic characteristics and explore the spatiotemporal clustering of confirmed cases of scrub typhus in mainland China at the county level, we used surveillance data on monthly numbers of reported confirmed scrub typhus cases from January 2006 to December 2012, routinely collected by the China Information System for Disease Control and Prevention (CISDCP) (https://sslvpn.cdpc.chinacdc.cn). The criteria for a probable case of scrub typhus include epidemiological exposure histories (travelling to an endemic area and contact with chiggers or rodents within three weeks before the onset of illness), clinical manifestations (such as high fever, lymphadenopathy, skin rash, and eschars or ulcers), and an agglutination titer ≥1∶160 in the Weil-Felix test using the OX_K_ strain of *Proteus mirabilis*. The case definition of confirmed scrub typhus must fulfil the above criteria for a probable case and also meet at least one of the laboratory criteria for confirmatory diagnosis: a fourfold or greater rise in serum IgG antibody titers between acute and convalescent sera detected by using indirect immunofluorescence antibody assay (IFA), detection of *O. tsutsugamushi* by polymerase chain reaction (PCR) in clinical specimens, or isolation of *O. tsutsugamushi* from clinical specimens [15].

A total of 27,391 confirmed cases of scrub typhus were reported during 2006–2012 in China, with males and females approximately equally affected (13,498 cases in males versus 13,893 cases in females). While sex-specific scrub typhus incidence from 2006 to 2010 did not change significantly over time, in 2011 and 2012 the incidence among females was significantly higher than that among males (p<0.001). People aged 40–49, 50–59, and 60–69 years comprised 18.06%, 21.36%, and 16.00% of cases respectively (Table 1). Annual average age-specific scrub typhus incidence varied, with the highest incidence of 0.66/100,000 in the 60–69 year-old age group and the lowest of 0.11/100,000 in the 10–19 year-old age group. Farmers had a higher incidence than non-farmers (χ^2^ = 1241.56, p<0.001). The proportion of scrub typhus cases occurring in farmers increased over the study period from 58.51% in 2006 to 69.33% in 2012, with a farmer to non-farmer (e.g., students, workers, and teachers) ratio of 1.43∶1 in 2006 and 2.26∶1 in 2012 (Cochran-Armitage trend test, z = 9.46, p<0.001).

**Table 1:** Characteristics of scrub typhus cases in mainland China, 2006–2012.

	No. (%) cases	Incidence (1/100,000)	OR^*^	95% CI^#^
**Sex**				
Male	13,498 (49.28)	0.29	Ref.	
Female	13,893 (50.72)	0.31	1.08	1.02–1.15
**Age group**				
<10	3,243 (11.84)	0.29	2.62	2.22–3.10
10-	1,398 (5.10)	0.11	Ref.	
20-	1,749 (6.39)	0.12	1.07	0.88–1.29
30-	2,863 (10.45)	0.20	1.83	1. 55–2.17
40-	4,946 (18.06)	0.33	3.04	2.60–3.56
50-	5,850 (21.36)	0.49	4.52	3.88–5.28
60-	4,382 (16.00)	0.66	6.08	5.19–7.13
≥70	2,960 (10.81)	0.63	5.77	4.88–6.82
**Farmers**				
No	8,877 (32.41)	0.16	Ref.	
Yes	18,514 (67.59)	0.50	3.13	2.93–3.35
**Year**				
2006	1,248(4.56)	0.10	Ref.	
2007	1,336(4.88)	0.10	1.07	0.99–1.16
2008	2,607(9.52)	0.20	2.09	1.95–2.23
2009	3,233(11.80)	0.25	2.59	2.43–2.77
2010	4,083(14.91)	0.31	3.27	3.07–3.49
2011	5,998(21.90)	0.46	4.81	4.52–5.11
2012	8,886 (32.44)	0.68	7.12	6.71–7.55

OR^*^: odds ratio; CI^#^: confidence interval; Ref.: reference category.

**Figure 1:**
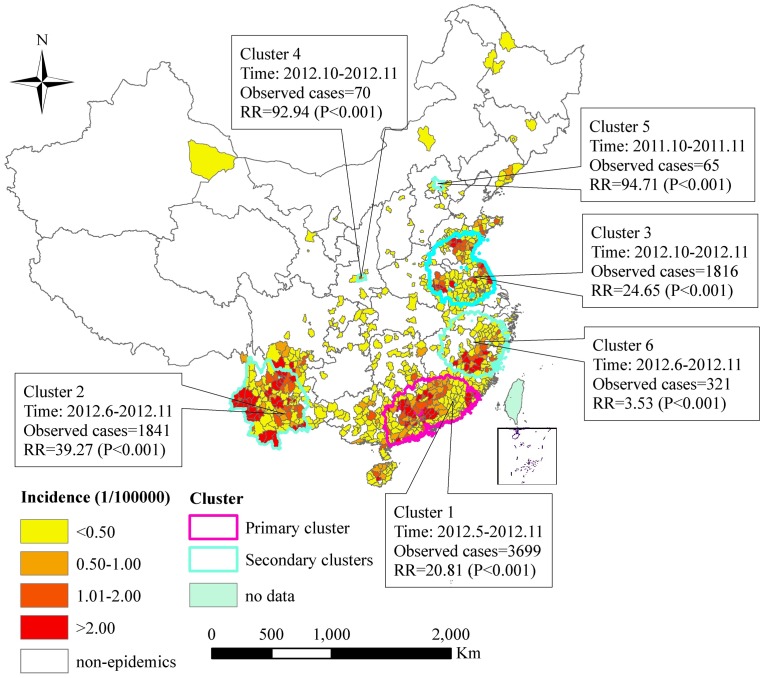
Annualized average incidence and space-time clusters of scrub typhus cases at the county level in China, 2006–2012.

The majority (81.22%) of confirmed cases of scrub typhus during 2006–2012 in China occurred yearly between July and November. Overall, the annual scrub typhus incidence had an increasing trend over the seven-year period (Cochran-Armitage trend test, z = 101.85, p<0.001). The number of confirmed scrub typhus cases peaked in 2012, when there were 7.12 times more reported cases than in 2006 (Table 1). The spatial distribution of scrub typhus showed that cases were mainly located in Southwest China, Southeast Coastal and Eastern regions (Figure 1). A total of 791 counties from 28 provinces have reported scrub typhus cases during the study period. Our results showed that the geographical distribution of scrub typhus has expanded, in that the number of counties with scrub typhus cases increased from 216 counties in 2006 to 574 counties in 2012, with an apparent increasing trend over time (Cochran-Armitage trend test, z = 18.14, p<0.001).

To define spatial autocorrelation in scrub typhus cases at the county level we used Global Moran's *I* (ArcGIS version 9.3, ESRI, Redlands, California), in which a negative value indicates a dispersed distribution and a positive value indicates a clustered distribution [16]. This analysis showed that scrub typhus cases were clustered in China, but that spatial correlation was weak (Moran's *I* = 0.08, p<0.01). To identify the location of spatiotemporal clusters, we used spatial scan statistics in SaTScan (version 9.1.1) [17]. A discrete Poisson model was assumed to estimate the relative risk (RR) of each cluster and the log likelihood ratio (LLR), to identify the primary cluster and secondary clusters. Significance of clusters was evaluated using 999 Monte Carlo replications, with a maximum circular scan window of 15% of the population at risk and 15% of the study period. The spatial reference was the geographical centre of each county and statistical significance of clusters was assessed using p-value<0.05. The results of this analysis identified a significant primary cluster (cluster 1) of scrub typhus cases located in 176 counties of Guangdong Province, Fujian Province, Jiangxi Province, and Guangxi Province, which occurred during May–November 2012 (RR = 20.81, p<0.01; Figure 1). Public health interventions to limit scrub typhus transmission, such as health promotion, environmental management, and rodent and vector control, should be prioritized in these regions. Our study also identified five secondary clusters (clusters 2–6). The secondary clusters were dispersed throughout the Southwest, East and Northeast of China (Figure 1). Cluster 2, located in 107 counties of Yunnan and Sichuan Provinces, occurred between June 2012 and September 2012 (RR = 39.27, p<0.01). Cluster 3, mainly located in 215 counties of Jiangsu, Anhui, and Shandong Provinces, occurred between October and November 2012 (RR = 24.65, p<0.01). Cluster 4, located in two counties of Shaanxi Province, occurred between October and November 2012 (RR = 92.94, p<0.01). Cluster 5, located in Pinggu District of Beijing Municipality, occurred between October and November 2011 (RR = 94.71, p<0.01). Cluster 6, mainly located in Fujian Province, occurred between October and November 2012 (RR = 3.53, p<0.01).

## Conclusion

The results of this study will be beneficial for informing future regional surveillance, patient management, and developing effective public health responses to scrub typhus, in that it demonstrated a rapid increase in case reports during 2006–2012 that was either due to significant geographical expansion of the disease beyond its previous natural foci or better physician awareness and improved diagnostics. Our results also showed that most scrub typhus cases occurred between July and November, which may be associated with increased exposure to infected chigger mites during the harvest season [18]. The disease was found to be prominent in elderly farmers, which may be associated with the rapidly changing demographics of rural areas in China [10]. In the past two decades, most younger males have left rural areas to work in the cities, and people engaging in agricultural activities are mainly more than 40 years old. Interestingly, the incidence among children aged less than ten years was higher than the 10–20 and 20–30 year-old age groups, which may be partly explained by an increased exposure opportunity for this age group during outdoor play.

More importantly, our study shows that scrub typhus remains an important public health problem in China, partly due to the lack of effective control of disease in persistent foci, which may have contributed to its emergence in newly identified foci. In that regard, this study showed a remarkable heterogeneity in the spatiotemporal distribution of scrub typhus cases in China. Scrub typhus cases are mainly located in areas of high population density in southeastern and southwestern China, indicating that many millions of people are at risk of infection. Importantly, this study identified a primary cluster located in 176 counties of Guangdong Province, Fujian Province, Jiangxi Province, and Guangxi Province, which is considered the main persistent foci of scrub typhus in China. These areas should be targeted by policy-makers and local service providers for the establishment of refined disease control guidelines, including local vector control, health education, and promotion campaigns. We also identified secondary high-risk clusters, comprising the newly identified foci of scrub typhus, including the capital Beijing, that resulted from the importation of infection from another province in 2007 [18].

The expansion of scrub typhus foci may be associated with factors of the physical environment and human behaviour, and future studies should be conducted to identify the role of these factors in the epidemiology of scrub typhus in the high-risk areas of China.
